# Language barriers and postoperative opioid prescription use after total knee arthroplasty

**DOI:** 10.1016/j.rcsop.2022.100171

**Published:** 2022-08-23

**Authors:** Kevin H. Nguyen, Aksharananda Rambachan, Derek T. Ward, Solmaz P. Manuel

**Affiliations:** a University of California, San Francisco School of Medicine, 533 Parnassus Ave, San Francisco, CA 94143, USA; b Division of Hospital Medicine, University of California, San Francisco, 521 Parnassus Ave, San Francisco, CA 94143, USA; c Department of Orthopaedic Surgery, University of California, San Francisco, 1500 Owens St, San Francisco, CA 94158, USA; d Department of Anesthesia and Perioperative Care, University of California, San Francisco, 521 Parnassus Ave, 4th Floor, San Francisco, CA 94143, USA

**Keywords:** Limited English proficiency, Opioids, Total knee arthroplasty, Health service inequities, Language-based disparities, Healthcare disparities

## Abstract

**Background:**

Patients with limited English proficiency (LEP) face difficulties in access to postoperative follow-up care, including post-discharge medication refills. However, prior studies have not examined how utilization of prescription pain medications after discharge from joint replacement surgeries differs between English proficient (EP) and LEP patients.

**Objective:**

This study explored the relationship between English language proficiency and opioid prescription refill requests after hospital discharge for total knee arthroplasty (TKA).

**Methods:**

This was an observational cohort study of patients ≥18 years of age who underwent TKA between January 2015 and December 2019 at a single academic center. LEP status was defined as not having English as the primary language and requesting an interpreter. Primary outcome variables included opioid pain medication refill requests between 0 and 90 days from discharge. Multivariable logistic regression modeling calculated the odds ratios of requesting an opioid refill.

**Results:**

A total of 2148 patients underwent TKA, and 9.8% had LEP. Postoperative pain levels and rates of prior opioid use did not differ between LEP and EP patients. LEP patients were less likely to request an opioid prescription refill within 30 days (35.3% vs 52.4%, p < 0.001), 60 days (48.7% vs 61.0%, p = 0.004), and 90 days (54.0% vs 62.9%, p = 0.041) after discharge. In multivariable analysis, LEP patients had an odds ratio of 0.61 of requesting an opioid refill (95% CI, 0.41–0.92, p = 0.019) within 30 days of discharge. Having Medicare insurance and longer lengths of hospitalization were correlated with lower odds of 0–30 days opioid refills, while prior opioid use and being discharged home were associated with higher odds of opioid refill requests 0–30 days after discharge for TKA.

**Conclusions:**

Language barriers may contribute to poorer access to postoperative care, including prescription medication refills. Barriers to postoperative care may exist at multiple levels for LEP patients undergoing surgical procedures.

## 1 Introduction

Surgical management of advanced osteoarthritis with total knee arthroplasty (TKA) has consistently improved pain, function, and symptoms of advanced joint arthritis.^1^ As the population ages and the demand for TKA procedures are expected to rise, there have been well-documented disparities in access to and outcomes after TKA for nonwhite minority populations relative to white majority populations in the United States.2., 3., 4., 5.

Compared to majority populations, racial minority groups were less likely to undergo TKA^6^^,^^7^ and have a greater risk of postoperative complications and readmission after TKA while deriving fewer patient-reported benefits from these procedures.8., 9., 10., 11., 12. It is unknown, however, to what degree these healthcare disparities are mediated or compounded by language barriers that may exist for vulnerable patient populations who do not speak English proficiently.

There has been recent interest in studying how limited English proficiency (LEP) is associated with differential healthcare outcomes. Despite national efforts to improve care for LEP patients, including the culturally and linguistically appropriate services (CLAS) guidelines in 2001 and the Patient Protection and Affordable Care Act in 2016, LEP patients continue to face healthcare disparities.^13^^,^^14^ Broadly, patients with LEP have worse access to care and poorer self-reported health compared to English proficient (EP) patients.^15^ Within the field of arthroplasty, LEP patients have longer lengths of hospitalization and are less likely to be discharged home.16., 17., 18. Postoperatively, LEP patients have also been found to have poorer functional outcomes and higher levels of postoperative pain.^19^ While it has been well documented that patients with LEP have poorer healthcare access and outcomes, further research is needed into the specific mechanisms through which language barriers faced by LEP patients affect surgical outcomes and their postsurgical recovery process.

Differences in postoperative outcomes based on English proficiency status may be partially due to language barriers that mediate reduced access to care and decreased utilization of prescription medications.^20^^,^^21^ Historically, up to 90% of patients undergoing TKA receive opioids for postoperative pain management, yet prior studies have not looked at differences in postoperative opioid prescription usage after TKA by English language proficiency.^22^^,^^23^ Identifying disparities in medication utilization after TKA can provide insight into barriers faced by vulnerable patient populations.

The purpose of this study is to investigate the relationship between LEP and requests for postoperative opioid prescription refills up to 90 days after hospital discharge for TKA. We hypothesize that LEP status is correlated with decreased rates of opioid prescription refills after TKA.

## 2 Methods

### 2.1 Participants

This retrospective cohort study analyzed electronic health record (EHR) information of all patients ≥18 years of age who underwent TKA between January 2015 and December 2019 at a single academic medical center located in a racially and linguistically diverse urban setting. These included primary TKA, revision TKA, elective cases, and urgent cases. Exclusion criteria included patients who died during the hospitalization. The affiliated academic institutional review board approved a waiver of consent.

### 2.2 Procedure

All TKA operations were performed by a single academic institution's orthopedic surgery service. TKA patients underwent standard total knee replacement using a variety of surgical techniques by one of five high-volume joint replacement specialists. The preoperative and postoperative multi-modal pain control protocols for patients were standardized between surgeons and adhered to evidence-based strategies for lower extremity arthroplasty. This included, unless medically contraindicated, preoperative dosing of a non-steroidal anti-inflammatory (meloxicam or celecoxib), acetaminophen, and gabapentin. Patients received procedural anesthesia through either spinal or general anesthesia with an adductor canal block. Intraoperatively a peri-articular injection was delivered consisting of ropivacaine, ketorolac, clonidine, and epinephrine, as described by Delury and colleagues.^24^ Postoperatively, patients were placed on around-the-clock acetaminophen, meloxicam, gabapentin at night, and as-needed doses of oxycodone. Patients were discharged with the same dosing and timing for the anti-inflammatory (meloxicam or celecoxib), acetaminophen, and gabapentin as they had inpatient. All knee arthroplasty patients were also discharged with a single standardized prescription for 40 pills of oxycodone 5 mg as-needed, without refill orders written for this initial opioid prescription. Protocols were adjusted for patients with chronic pain and higher opioid requirements.

### 2.3 Study design

The primary predictor variable in this analysis was English proficiency status, where LEP was defined as self-reporting a non-English primary language and requesting interpreter services at the time of admission. For patients whose extracted demographic data revealed only one of the criteria (non-English primary language or request for interpretation), LEP status was confirmed through manual chart review. Primary outcome variables included opioid pain medication refill requests between 0 and 30 days, 0–60 days, and 0–90 days from discharge after TKA.

Demographic information, preoperative health status, perioperative information, discharge disposition, and post-discharge opioid orders were obtained from patients' EHR. Demographic information included age, self-reported gender, self-reported race/ethnicity, primary spoken language, primary insurance type, and residential zip code. Sensitivity analysis assessing age as a categorical versus continuous variable revealed no changes that impacted our study conclusions. Patients' race/ethnicity were categorized as non-Hispanic white, Hispanic, Asian American and Pacific Islander (AAPI), Black, Indigenous, or other. “Other” race/ethnicity was defined as patients who self-identified their race and ethnicity as either unknown, declined, or other. We acknowledge that these racial and ethnic categories reflect social rather than biological or genetic groups.^25^ The primary language was categorized as English, Spanish, Chinese (including Mandarin, Cantonese, and Toishanese), Russian, or other languages. Insurance types included private commercial or employer-provided insurance, Medicaid, or Medicare. Median household income by 5-digit USPS zip code was obtained using 2019 United States Census Bureau data.^26^ Preoperative health metrics included the American Society of Anesthesiologists (ASA) rating, body mass index (BMI), smoking status, and prior opioid use. ASA rating is a classification system that assesses patients' preoperative fitness and is a proxy for comorbidity burden.^27^ Perioperative and postoperative variables included preoperative pain level, 1-h postoperative pain level, total length of hospitalization, and discharge disposition. Preoperative and 1-h postoperative pain levels were patient-reported scores based on a 0–10 scale. Discharge disposition was categorized as either being discharged home (with self-care or home health) or other inpatient dispositions (including skilled nursing facilities, acute rehabilitation facilities, and acute care hospitals).

### 2.4 Statistical analysis

Bivariate analyses comparing EP and LEP patient characteristics were performed using a *t*-test for continuous variables and Fisher's exact test for categorical variables. Multivariable logistic regression modeling was performed to calculate the odds ratio of opioid refill requests 0–30 days after discharge. Covariates, chosen a priori based on prior TKA literature and availability in the dataset, included age, gender, BMI, ASA rating, median income based on residential zip code, insurance type, length of hospitalization, history of preoperative opioid use, 1-h postoperative pain scores, and discharge disposition.28., 29, 30. Race/ethnicity was excluded from the multivariable logistic regression model due to high collinearity between race/ethnicity categories with LEP status. Statistical significance was set to be a two-sided p-value <0.05. All statistical analyses were performed using Stata version 16.1 (StataCorp).

The reporting of this study conforms to the Strengthening the Reporting of Observational Studies in Epidemiology (STROBE) guidelines.^31^

## 3 Results

A total of 2148 patients underwent TKA between January 2015 and December 2019. Overall, 9.8% (211) of these patients were classified as having LEP. Compared to EP patients, patients with LEP were more likely to be female (73.5% vs. 58.3%, p < 0.001), older (68.9 ± 12.0 years vs. 64.8 ± 10.0 years, p < 0.001), less likely to have private insurance (13.3% vs. 39.6%, p < 0.001), and live in a zip code with lower median household income ($92,707 ± $35,181 vs. $104,315 ± $41,442, p < 0.001). Among LEP patients, Hispanic (42.7%) and AAPI (37.4%) were the most common race/ethnicity groups, while the majority of EP patients identified as non-Hispanic white (70.5%). LEP patients' most common primary spoken languages were Spanish (42.7%) and Chinese (25.1%). There were no differences between LEP and EP patients in ASA rating, BMI, or smoking status (Table 1). Postoperatively, LEP patients, relative to EP patients, had a longer length of hospitalization (2.79 ± 2.63 days vs. 2.29 ± 1.82 days, p < 0.001) and were less likely to be discharged home (72.0% vs. 82.4%, p = 0.001) (Table 1).

**Table 1:** Demographics and perioperative characteristics by English proficiency for knee arthroplasty patients.

	Total sample (N = 2148)	English proficient (n = 1937)	Limited English proficiency (n = 211)	P value
**Gender, % (n)**^a^				**<0.001**
Female	59.8% (1284)	58.3% (1129)	73.5% (155)	
Male	40.2% (864)	41.7% (808)	26.5% (56)	
**Age, years % (n)**^a^				**<0.001**
<50	6.2% (132)	6.2% (119)	6.2% (13)	
50–59	19.1% (409)	20.1% (388)	10.0% (21)	
60–69	38.9% (834)	39.8% (769)	30.8% (65)	
70–79	30.4% (651)	29.7% (573)	37.0% (78)	
80+	5.5% (117)	4.3% (83)	16.1% (34)	
**Race/Ethnicity, % (n)**^a^				**<0.001**
Non-Hispanic White	64.9% (1393)	70.5% (1366)	12.8% (27)	
Hispanic	11.4% (244)	8.0% (154)	42.7% (90)	
AAPI	10.4% (224)	7.5% (145)	37.4% (79)	
Black	7.7% (166)	8.6% (166)	0% (0)	
Indigenous	0.7% (14)	0.7% (14)	0% (0)	
Other	5.0% (107)	4.7% (92)	7.1% (15)	
**Language Spoken, % (n)**^a^				**<0.001**
English	90.1% (1936)	100% (1936)	0% (0)	
Spanish	4.2% (90)	0% (0)	42.7% (90)	
Chinese	2.5% (53)	0% (0)	25.1% (53)	
Russian	0.8% (17)	0% (0)	8.0% (17)	
Other Non-English	2.4% (51)	0% (0)	24.2% (51)	
**Primary Insurance Type, % (n)**^a^				**<0.001**
Private	37.0% (794)	39.6% (766)	13.3% (28)	
Medicaid	11.8% (253)	9.7% (188)	30.8% (65)	
Medicare	51.3% (1101)	50.8% (983)	55.9% (118)	
**Zip Code Median Income, USD (SD)**^b^	$103,164 ($41,004)	$104,315 ($41,442)	$92,707 ($35,181)	**<0.001**
**ASA Rating, % (n)**^a^				0.565
ASA 1	3.9% (84)	4.1% (79)	2.4% (5)	
ASA 2	66.6% (1430)	66.3% (1284)	69.2% (146)	
ASA 3	29.1% (625)	29.2% (566)	28.0% (59)	
ASA 4	0.4% (9)	0.4% (8)	0.5% (1)	
**BMI, kg/m**^**2**^**(SD)**^b^	30.3 (6.0)	30.4 (6.0)	30.0 (5.5)	0.465
**Smoker, % (n)**^a^				0.118
No	98.0% (2106)	97.9% (1896)	99.5% (210)	
Yes	2.0% (42)	2.1% (41)	0.5% (1)	
**Length of Stay, days (SD)**^b^	2.3 (1.9)	2.29 (1.82)	2.79 (2.63)	**<0.001**
**Discharge Disposition, % (n)**^a^				**0.001**
Other Inpatient	18.6% (399)	17.6% (340)	28.0% (59)	
Home	81.4% (1748)	82.4% (1596)	72.0% (152)	

Data are presented as mean (SD) for continuous measures and % (n) for categorical measures. ^a^Fisher's exact test ^b^Two sample t-test. Abbreviations: AAPI, Asian-American & Pacific Islander; ASA, American Society of Anesthesiologists; BMI, body mass index.

**Fig. 1:**
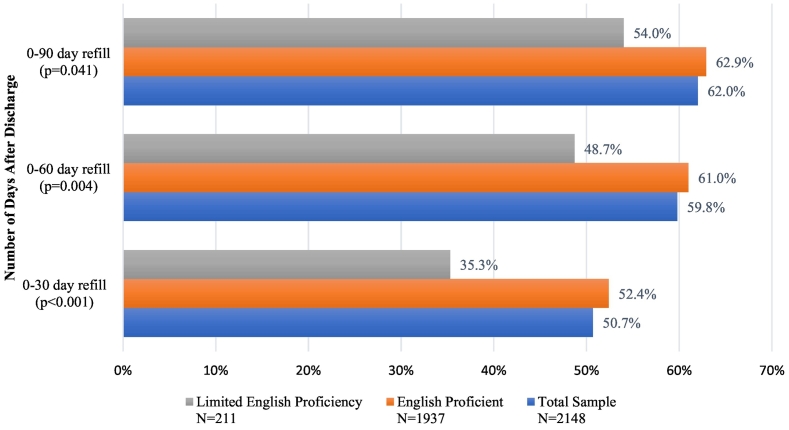
Rates of opioid prescription refills after total knee arthroplasty.

Self-reported preoperative and 1-h postoperative pain levels did not differ between LEP and EP patients. The proportions of patients who reported prior opioid use also did not differ between LEP and EP patients (Table 2). In bivariate analyses, LEP patients were less likely to request opioid prescription refills in the first 30 days (35.3% vs 52.4%, p < 0.001), 60 days (48.7% vs 61.0%, p = 0.004), and 90 days (54.0% vs 62.9%, p = 0.041) after discharge for TKA (Fig. 1).

**Table 2:** Pain levels and prior opioid use.

	Total sample (N = 2148)	English proficient (n = 1937)	Limited English proficiency (n = 211)	P value
Preoperative Pain Level, mean (SD)^a^	3.55 (3.19)	3.52 (3.15)	3.79 (3.54)	0.309
1 h Postoperative Pain Level, mean (SD)^a^	2.02 (3.20)	2.02 (3.21)	2.03 (3.08)	0.956
Prior Opioid Use, % (n)^b^	35.9% (771)	36.5% (707)	30.3% (64)	0.082

a Two sample t-test. ^b^Fisher's exact test.

In multivariate logistic regression models, being classified as LEP was significantly associated with lower odds of requesting an opioid prescription refill 0–30 days after discharge (OR: 0.61, CI: 0.41–0.92, p = 0.019). Having Medicare insurance (OR: 0.62, CI: 0.47–0.82, p = 0.001) and longer lengths of hospitalization (OR: 0.91, CI: 0.84–0.98, p = 0.017) were also associated with decreased odds of requesting an opioid prescription 0–30 days after discharge. On the other hand, prior opioid use (OR: 1.40, CI: 1.09–1.82, p = 0.009) and being discharged to home (OR: 3.20, CI: 2.28–4.48, p < 0.001) were associated with increased odds of requesting an opioid prescription refill 0–30 days after discharge. In this multivariate model, age, gender, BMI, ASA rating, median household income, and 1-h postoperative pain level were not statistically significantly associated with either increased or decreased odds of 0–30 day opioid refills (Table 3).

**Table 3:** Adjusted odds ratios of opioid refills within 30 days after discharge.

	Adjusted coefficient (OR)	P value	[95% CI]	
English proficiency				
English Proficient	1			
Limited English Proficiency	0.61	**0.019**	0.41	0.92
**Age**				
<50	1			
50–59	0.74	0.267	0.44	1.25
60–69	1.13	0.644	0.68	1.85
70–79	0.90	0.709	0.53	1.55
80+	0.47	0.053	0.22	1.01
**Gender**				
Male	1			
Female	1.12	0.344	0.89	1.41
**BMI**	0.99	0.506	0.97	1.01
**ASA Rating**				
ASA 1	1			
ASA 2	1.22	0.502	0.69	2.16
ASA 3	1.10	0.758	0.60	2.03
**Median Household Income**	1.00	0.307	0.99	1.00
**Primary Insurance Type**				
Private Insurance	1			
Medicaid	1.20	0.377	0.80	1.78
Medicare	0.62	**0.001**	0.47	0.82
**Length of hospitalization**	0.91	**0.017**	0.84	0.98
**Prior Opioid Use**				
No	1			
Yes	1.40	**0.009**	1.09	1.82
**1h Postoperative Pain Level**	1.01	0.712	0.97	1.05
**Discharge Disposition**				
Other Inpatient	1			
Home	3.20	**<0.001**	2.28	4.48

Abbreviations: OR, odds ratio; CI, confidence interval; BMI, body mass index; ASA, American Society of Anesthesiologists.

LEP was not independently associated with significant differences in opioid prescription refill requests within the first 60 days and first 90 days after discharge in multivariate models after adjusting for age, gender, BMI, ASA rating, median household income, insurance type, hospitalization length, prior opioid use, 1-h postoperative pain level, and discharge disposition.

## 4 Discussion

This study aimed to explore the relationship between English proficiency status and post-discharge opioid prescription refill requests after TKA. Even after adjusting for potential confounding factors, LEP patients were found to be less likely to request opioid refills up to 30 days after discharge for TKA compared to EP patients, despite the lack of differences in preoperative or 1-h postoperative pain levels. This novel relationship between language status and postoperative opioid medication refill request rates adds to the current understanding of disparities in postoperative prescription medication utilization and postoperative care access for patients with LEP. These findings are consistent with a prior study by Schwartz et al. that found that LEP trauma patients are less likely to receive opioid prescriptions at discharge.^32^ Our study shows that differential access to postoperative opioids also exists for patients with LEP undergoing TKA and that this difference cannot be explained by confounding factors such as higher postoperative pain levels or history of preoperative opioid use, which are known strong predictors of postoperative opioid use.^33^^,^^34^

These findings of differential postoperative refill request rates are also consistent with studies that observed that LEP patients have less access to postoperative care than EP patients. A study by Khorgami et al. found that language differences are associated with decreased attendance rates for postoperative follow-up appointments.^35^ In addition, language barriers are associated with higher rates of prescription medication nonadherence, partially due to language discordancy affecting the quality of patient-provider communication.36., 37., 38.

This study's findings further add to the literature that highlights disparities in pain management for racial and ethnic minority patients.^39^ Specifically, it has been well documented that opioid prescriptions are less likely to be prescribed for nonwhite patient populations despite similar pain levels as white patients.^40^, 41., 42. However, while the relationship between patient race or ethnicity and opioid prescription patterns has been well described, less is known about how English proficiency levels are associated with opioid prescription rates. This study suggests that in addition to provider bias, patient preferences, and socioeconomic obstacles, language barriers may also explain this differential access to postoperative pain control prescriptions.^43^^,^^44^

Language barriers in TKA have also been shown to be associated with worse TKA outcomes, including longer lengths of hospitalization and lower rates of home discharges,^45^ which is consistent with this study's patient population's postoperative outcomes. This study's findings add to this narrative of inferior postoperative care after TKA for LEP patients by suggesting that language barriers may result in differential access to care after discharge. The differences in access to opioid prescriptions exist for up to 3 months after discharge and suggest there may be underlying multifactorial causes.

Prior literature suggests inadequate access to professional interpreters as a mediator of postoperative healthcare disparities for LEP patients. This may be especially true in orthopedic surgery, where there is a heavy reliance on ad hoc interpreters.^46^ Insufficient access to professional interpreter services can lead to a poorer understanding of discharge medication instructions and adverse medication effects,47., 48., 49. which may contribute to higher risks of nonadherence to new post-discharge medications.^50^ On the other hand, reliable access to professional interpreters and language concordant care for LEP patients is associated with improved pain control and a better understanding of postoperative pain medications after joint replacement surgery.^51^^,^^52^

This study's observed disparity in postoperative opioid prescription refill rates may also be partially due to barriers at the pharmacy level. For example, it has been shown that pharmacy trainees lack the confidence to communicate with LEP patients regarding proper opioid usage and risks.^53^ Furthermore, compared to white-predominant communities, the majority of nonwhite neighborhood pharmacies are less likely to stock opioids,^54^ and LEP patients are also less likely to use remote refilling systems.^55^ All these factors may make it more difficult for LEP patients to be properly informed and access postoperative prescriptions through their local pharmacies.

While our results suggest that language barriers likely negatively affect LEP patients' access to postoperative medications, we acknowledge that disparities in postoperative care are complex and multifactorial, including, but not limited to, medication prescribers' own biases, cultural preferences, socioeconomic barriers, and perceived pain levels.56., 57., 58.

This study is subject to several limitations. First, this study relies on refill requests, rather than pharmacy dispensing data, as an indicator for access to postoperative prescription medications. Dispensing data would be a more direct measure of whether patients receive refills. However, it is reasonable to infer that decreased refill requests likely correlate to decreased prescription pickups rates. Secondly, while this study's database had thorough data on opioid prescriptions for 90 days after discharge, oral morphine equivalence (OME) received and taken by each patient would have been a more granular measurement of postoperative opioid usage. Future studies can extrapolate OME data from pharmacy records to further explore the relationship between LEP and post-discharge opioid access. Also, this study design did not allow for collecting qualitative data from the stakeholders, namely the surgeons and the patients. Future projects should aim to interview patients and providers to elucidate provider- and patient-specific factors that affect opioid prescribing and usage after discharge for TKA. Lastly, this project solely focused on opioid prescriptions after discharge and did not assess the utilization of non-opioid multi-modal pain medications that were a part of their regimen. However, it is known that most TKA patients continue to rely heavily on opioid prescriptions for postoperative pain control after discharge.^23^ In addition, all patients in this study were discharged from the same surgical service at a single institution, which decreases the variance in other non-opiate pain medications they may have received upon discharge.

## 5 Conclusions

This retrospective cohort study found that patients with limited English proficiency are less likely than English proficient patients to request opioid refills after discharge for TKA. These findings suggest that language barriers may adversely affect patients' access to adequate postoperative care, including prescription medications, which may have downstream effects on recovery and quality of life after surgery. These findings support further research and investment into the infrastructure and systems that support perioperative and postoperative care for patients with limited English proficiency.

## Funding

This study was supported through grant funds from the Hellman Family Foundation and the American Society of Anesthesiologists Committee on Professional Diversity.

## CRediT authorship contribution statement

**Kevin H. Nguyen:** Methodology, Software, Formal analysis, Investigation, Data curation, Visualization, Writing – original draft. **Aksharananda Rambachan:** Validation, Writing – review & editing. **Derek T. Ward:** Conceptualization, Methodology, Project administration, Supervision, Writing – review & editing. **Solmaz P. Manuel:** Conceptualization, Methodology, Formal analysis, Investigation, Project administration, Supervision, Writing – review & editing.

## Declaration of Competing Interest

Dr. Ward has received consulting stipends from Depuy (Johnson & Johnson) and there are no other conflicts to report.
